# Immune Response of an Oral *Enterococcus faecalis* Phage Cocktail in a Mouse Model of Ethanol-Induced Liver Disease

**DOI:** 10.3390/v14030490

**Published:** 2022-02-27

**Authors:** Beatriz Garcia Mendes, Yi Duan, Bernd Schnabl

**Affiliations:** 1 Department of Medicine, University of California San Diego, La Jolla, San Diego, CA 92093, USA; beatriz.mendes@ufsc.br (B.G.M.); yid003@health.ucsd.edu (Y.D.); 2 Department of Clinical Analysis, Federal University of Santa Catarina, Florianópolis 88040-900, Brazil; 3 Department of Medicine, VA San Diego HealthCare System, San Diego, CA 92161, USA

**Keywords:** alcoholic hepatitis, *Enterococcus faecalis*, phage therapy, immune response, phage translocation, host range

## Abstract

Cytolysin-positive *Enterococcus faecalis* (*E. faecalis*) cause more severe alcohol-associated hepatitis, and phages might be used to specifically target these bacteria in a clinical trial. Using a humanized mouse model of ethanol-induced liver disease, the effect of cytolytic *E. faecalis* phage treatment on the intestinal and liver immune response was evaluated. The observed immune response was predominantly anti-inflammatory and tissue-restoring. Besides, live phages could be readily recovered from the serum, spleen, and liver following oral gavage in ethanol-fed mice. We also isolated 20 new phages from the sewage water; six of them exhibited a relatively broad host range. Taken together, the oral administration of cytolytic *E. faecalis* phages leads to the translocation of phages to the systemic circulation and appears to be safe, following chronic-binge ethanol administration. A cocktail of three phages covers the majority of tested cytolysin-positive *E. faecalis* strains and could be tested in a clinical trial.

## 1. Introduction

Alcohol-associated hepatitis is a severe and life-threatening form of alcohol-associated liver disease, with no available effective pharmacotherapy options. The presence of cytolysin-positive *Enterococcus faecalis* in the fecal microbiome was associated with liver disease severity and mortality in patients with alcohol-associated hepatitis [1], but the same correlation was not found in patients with non-alcoholic fatty liver disease (NAFLD) [2]. Treatment with bacteriophages targeting cytolytic *E. faecalis*, administered orally, reduced the translocation of the toxin to the liver and attenuated ethanol-induced liver injury, steatosis, and inflammation in humanized mice [1]. This indicates that phage treatment might precisely modify the intestinal microbiota in patients with alcohol-associated hepatitis, thereby improving clinical outcomes and decreasing mortality.

Before the initiation of a clinical trial, the safety of phage therapy for a severely sick patient population with alcohol-associated hepatitis must be addressed. It is important to assess the host immune response upon oral phage administration. Phages can migrate across the mucus layer, cross the gut epithelial barrier by either transcytosis or peptide-guided transport and subsequently interact directly with cells of both the innate and adaptive immune system [3,4,5,6]. That is important as patients with chronic alcohol consumption have a gut barrier dysfunction, allowing the translocation of microbial products and viable microbes from the intestinal lumen to the systemic circulation [7]. The time of phage persistence in the host’s body and their concentration in a particular organ strongly depends on the absence or presence of their target bacteria. Phages might modulate the immune response, contributing to immune homeostasis in the gastrointestinal tract [8,9]. Additionally, phages can interact with humoral immunity, which results in the production of neutralizing antibodies. These interactions play an important role in removing bacteriophages from higher organisms, such as in mice, rats, and humans [10]. Moreover, while some phages can only infect one or a few bacterial strains, other phages can infect many species or even bacteria from different genera and display a large diversity of target bacteria [11,12]. Therefore, the biology of phages and their host range are key properties for phage therapy.

Here, we assessed the immune response by monitoring the transcription of specific genes and the clearance of phages from the host in a preclinical mouse model. Using a humanized mouse model of ethanol-induced liver disease, we evaluated the effect of the oral administration of cytolytic *E. faecalis* phages on the intestinal and liver immune response. We used PBS as a placebo, but not control phages. Additionally, we assessed translocation of orally administered phages from the gut to organs and systemic circulation following ethanol administration. Finally, given the limited host range of phages, new phages were isolated from sewage water, and the host range was determined against cytolytic *E. faecalis* strains from patients with alcohol-associated liver disease. Increasing the number of available phages increases the chances that they recognize other cytolytic *E. faecalis* isolated from additional patients with alcohol-associated hepatitis in a clinical trial.

## 2. Materials and Methods

### 2.1. Murine Model and Phage Application

Initially, germ-free C57BL/6NTac mice were obtained from Taconic Biosciences (Rensselaer, NY, USA) [13] and then bred at the University of California San Diego. Fecal transplantation was performed into germ-free mice using a cytolysin-positive stool sample from a human donor with alcohol-associated hepatitis [1]. Colonized female and male mice (age 9–12 weeks) were placed on a chronic-binge ethanol diet (NIAAA model) as previously described [14].

Gnotobiotic mice were fed with the Lieber-DeCarli diet to evaluate the immune response post phage treatment. The caloric intake from ethanol was 0% on days 1–5 and 36% from day 6 until the end of the study period. On day 16, mice were gavaged with a single dose of ethanol (5 g/kg body weight) in the early morning and euthanized 9 h later. The three phages (Ef 2.1, Ef 2.2, and Ef 2.3) against cytolysin-positive *E. faecalis* used in this experiment were purified by gradient centrifugation as previously described [1], and 100 μL of the phage cocktail (10^10^ PFUs) was gavaged to the mice one day before the ethanol binge. Treatment using PBS without phage cocktail was used as a control.

The same procedure was performed at different time points in order to evaluate phage translocation from the gut to extraintestinal organs and systemic circulation: (a) phages were administrated one day before the ethanol binge, and mice were euthanized 1 h after it; (b) phages were administrated 1 h after the ethanol binge, and mice were euthanized 1 h following phage administration; (c) ethanol binge was done 1 h before phages were administered, and mice were euthanized 5 min following phage administration; (d) phage cocktail was administrated one day before the ethanol binge, then the mice were euthanized one day after it; (e) ethanol binge was done one hour before phages administration, and the mice were euthanized one day later.

The study was conducted according to the guidelines of the Declaration of Helsinki and approved by the Institutional Animal Care and Use Committee of UCSD (protocol S09042, date of approval 21 January 2009, and current expiration date 21 April 2023).

### 2.2. Intestinal and Hepatic Immune Response

We investigated the intestinal and the liver immune response after phage administration by assessing the expression of genes encoding cytokines and chemokines. RNA was extracted from mouse tissues (jejunum, ileum, colon, and liver), and cDNAs were generated as previously described [1]. Mouse-gene expression was determined with Sybr Green (Bio-Rad Laboratories, Hercules, CA, USA) using ABI StepOnePlus real-time qPCR system. All primers used in this study are listed in Table 1. The qPCR value of mouse genes was normalized to 18S.

### 2.3. Phage Translocation

After full anesthesia with an i.p. injection of ketamine/xylazine, the chest cavity was opened, and venous blood was taken from the inferior vena cava. The blood was centrifuged at 3000× *g*, at 4 °C, for 15 min to obtain serum. Then, the fecal pellet was collected from the colon, added to 500 µL of PBS, homogenized, and centrifuged for 3 min at room temperature. The supernatant was transferred into a new tube, and the pellet was discarded. The liver and spleen were removed, added to 1 mL of PBS, and homogenized. 100 µL of each sample obtained without dilution, except for fecal samples that were serially diluted, were mixed with 100 µL of *E. faecalis* culture and then added to BHI broth top agar (0.5% agar) and poured over a BHI plate (1.5% agar). The plates were incubated overnight, and the number of plaques was recorded.

### 2.4. Bacteriophage Isolation

Novel *E. faecalis* phages were isolated from untreated raw sewage water obtained from North City Water Reclamation Plant in San Diego. Raw sewage water (50 mL) was centrifuged at 8000× *g* for 1 min at room temperature to pellet large particles. The supernatant was passed through a 0.45-μm, and then a 0.2-μm syringe filter (PES membrane, Whatman, Maidstone, UK). One hundred microliters of the clarified sewage water were mixed with 100 μL overnight *E. faecalis* culture, added to BHI broth top agar (0.5% agar), and poured over a BHI plate (1.5% agar). After overnight growth at 37 °C, the resulting plaques were recovered using a sterile pipette tip and added to 500 μL of PBS. Phages were replaqued on *E. faecalis* three more times to ensure that they were clonal isolates. High-titer phage stocks were propagated by plaquing four plates using 100 μL of the phage mixed with 100 μL overnight *E. faecalis* culture, added to BHI broth top agar (0.5% agar), and then poured over a BHI plate (1.5% agar). The plates were harvested using 3 mL of BHI medium to remove only the upper overlay agar layer; the lysates obtained were combined and centrifuged at 10,000× *g* for 3 min at room temperature, to remove the remaining bacterial cells and other debris. The supernatant was then filtered through a 0.2-μm syringe filter (Whatman, PES membrane) and kept at 4 °C until use.

For all isolated phages, 10 mL of lysates were treated with 10 μg/mL of DNase and 10 μg/mL of RNase at 37 °C for 1 h. Then, they were precipitated by adding 1 M NaCl and 10% (*w*/*v*) polyethylene glycol 8000 (PEG 8000) and incubated at 4 °C overnight. Precipitated phages were then pelleted by centrifugation at 10,000× *g* for 10 min at 4 °C and resuspended in 500 μL of resuspension solution containing 5 mM MgSO4. Phage DNA was then extracted using Promega Wizard DNA Clean-up kit (Promega, Madison, WI, USA). Purified DNAs were resuspended in 100 μL of RNase-free water, and DNA concentrations were measured with a NanoDrop ND-1000 Spectrophotometer (Thermo Fisher Scientific, Waltham, MA, USA). A total of 70 ng of the phage DNA was digested by each of the restriction enzymes (BamHI, HindIII, NdeI, PstI) for 2 h at 37 °C. The DNA cleavage patterns were compared with each other to allow the distinction of the isolated phages.

### 2.5. Host Range Determination

*E. faecalis* strains isolated from the feces of cytolysin-positive patients with alcohol-associated hepatitis were cultured overnight at 37 °C in BHI medium. One hundred microliters of the bacterial culture were mixed with 100 µL of the dilutions obtained from the different phages isolated and then added to BHI broth top agar (0.5% agar) and poured over a BHI plate (1.5% agar). The plates were incubated overnight, and the number of plaques was recorded. The efficiency of plating (EoP) was then calculated for each phage. EoP = PFUs using the specific bacterial strain/PFUs using the original host strain. For phages #20, #21, #23, #27–#29, the PFUs were about 10^7^–10^8^ using the original host bacterial strain; for all other phages, the PFUs were about 10^9^–10^10^ using the original host bacterial strain.

### 2.6. Statistical Analysis

Results were expressed as mean ± s.e.m. For mouse studies, the significance of multiple groups was evaluated using one-way ANOVA with Tukey’s post hoc test. Statistical analyses were performed using GraphPad Prism v.6.01. A value of *p* < 0.05 was considered to be statistically significant.

## 3. Results

### 3.1. Intestinal and Hepatic Immune Response following Oral Phage Administration

**Figure 1:**
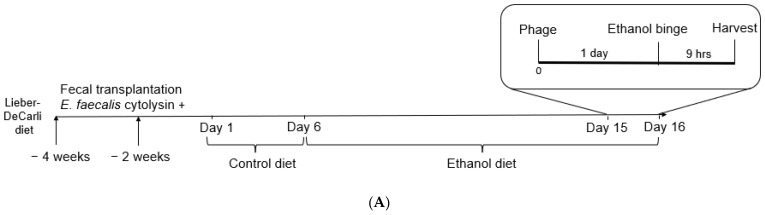

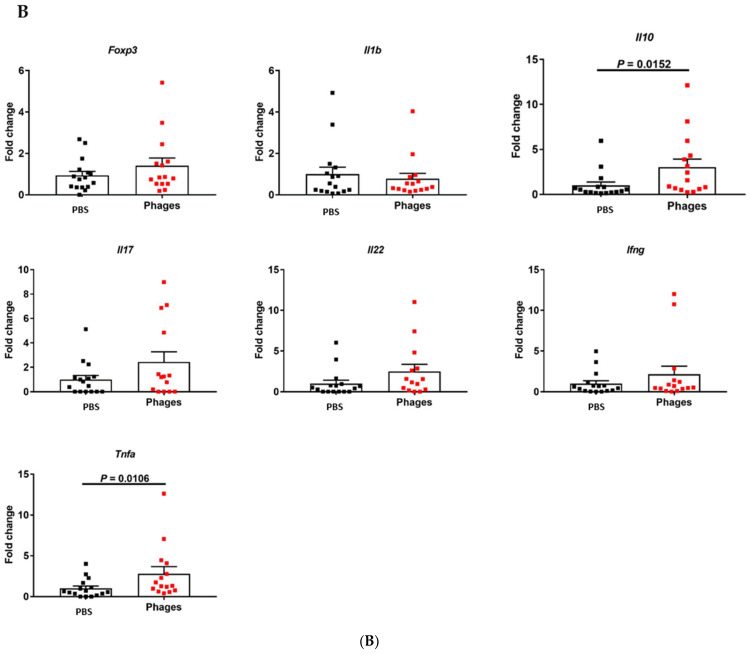
Effect of phage treatment on the immune response in the jejunum. C57BL/6 germ-free mice were colonized with feces from a cytolysin-positive patient with alcohol-associated hepatitis. Mice were then fed with a chronic-binge ethanol diet and gavaged with a phage cocktail that targets cytolytic *E. faecalis* (10^10^ PFUs) or PBS one day before an ethanol binge. (**A**) Experimental set-up; (**B**) gene expression in the jejunum. Results were expressed as mean ± s.e.m; *n* = 15 for the phage treatment group, and *n* = 16 for the PBS group.

Humanized mice transplanted with cytolysin-positive stool samples from patients with alcohol-associated hepatitis were subjected to the chronic-binge ethanol feeding model. Phages targeting cytolytic *E. faecalis* were orally gavaged one day before the ethanol binge (Figure 1A). As we have shown before, treatment with phages against cytolytic *E. faecalis* decreased fecal amounts of *Enterococcus* and reduced hepatic levels of cytolysin [1]. Phage treatment significantly increased interleukin-10 (*Il10*) and tumor necrosis factor-alpha (*Tnfa*) gene expression in the jejunum while Forkhead box P3 (*Foxp3*), a transcription factor for regulatory T cells, *Il1b, Il17, Il22*, and interferon-gamma (*Ifng*) were not significantly changed (Figure 1B).

**Figure 2:**
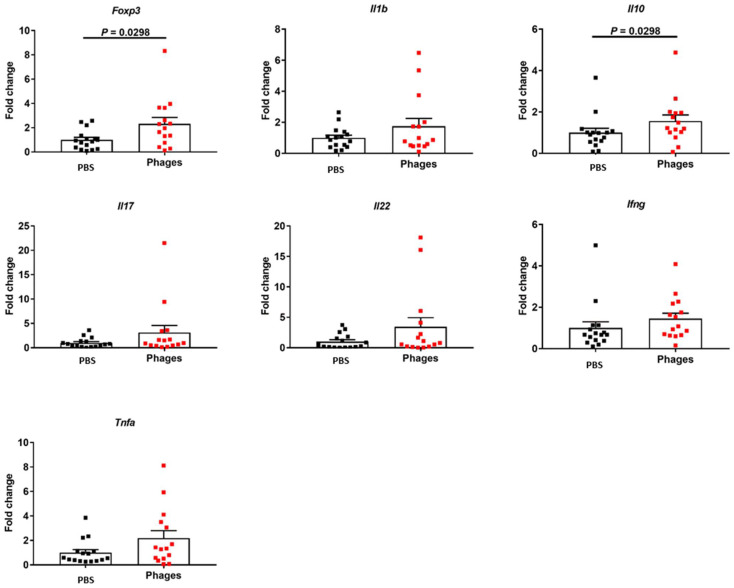
Effect of phage treatment on the immune response in the ileum. C57BL/6 germ-free mice were colonized with feces from a cytolysin-positive patient with alcohol-associated hepatitis. Mice were then fed with a chronic-binge ethanol diet and gavaged with a phage cocktail that targets cytolytic *E. faecalis* (10^10^ PFUs) or PBS one day before an ethanol binge. Gene expression in the ileum. Results were expressed as mean ± s.e.m; *n* = 15 for the phage treatment group, and *n* = 16 for the PBS group.

In the ileum, there was an increase in *Foxp3* and *Il10* gene expressionfollowing phage treatment, as compared with the negative control. The expression of other cytokines and chemokines was not changed (Figure 2).

**Figure 3:**
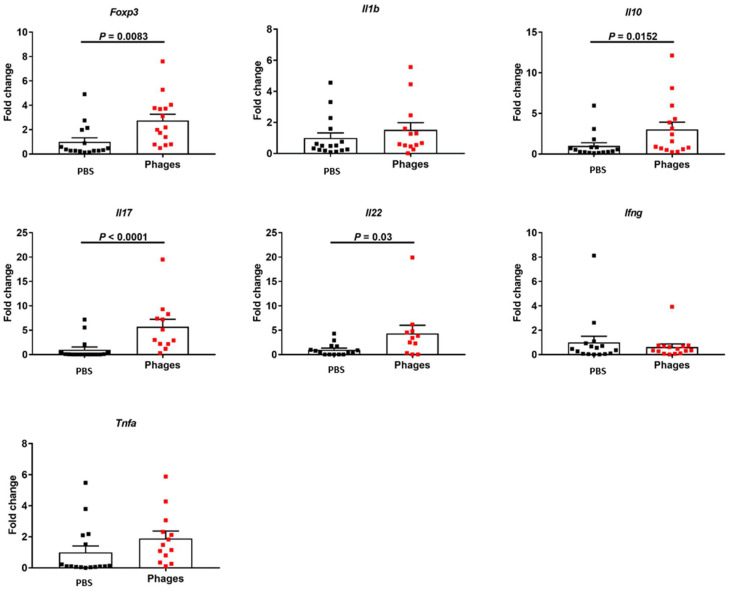
Effect of phage treatment on the immune response in the colon. C57BL/6 germ-free mice were colonized with feces from a cytolysin-positive patient with alcohol-associated hepatitis. Mice were then fed with a chronic-binge ethanol diet and gavaged with a phage cocktail that targets cytolytic *E. faecalis* (10^10^ PFUs) or PBS one day before an ethanol binge. Gene expression in the colon. Results were expressed as mean ± s.e.m; *n* = 15 for the phage treatment group, and *n* = 16 for the PBS group.

In the colon, *Foxp3, Il10, Il17*, and *Il22* showed higher gene expression levels following phage treatment in comparison with the vehicle group (Figure 3).

**Figure 4:**
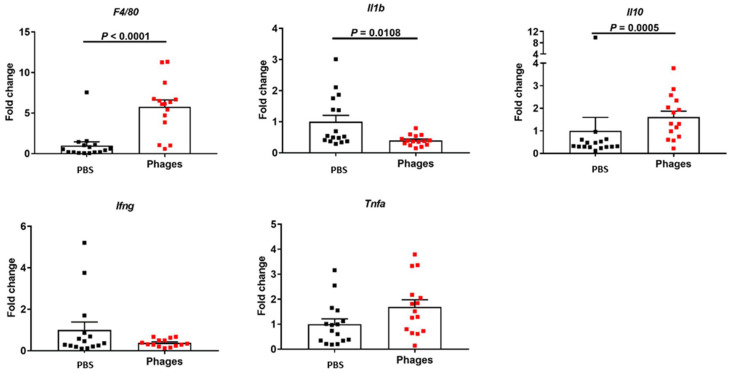
Effect of phage treatment on the liver immune response. C57BL/6 germ-free mice were colonized with feces from a cytolysin-positive patient with alcohol-associated hepatitis. Mice were then fed with a chronic-binge ethanol diet and gavaged with a phage cocktail that targets cytolytic *E. faecalis* (10^10^ PFUs) or PBS one day before an ethanol binge. Hepatic gene expression. Results were expressed as mean ± s.e.m; *n* = 15 for the phage treatment group, and *n* = 16 for the PBS group.

In addition, we also checked the immune response in the liver. Ethanol-fed mice treated with phages had higher levels of mRNAs encoding *F4/80*, a marker for resident liver macrophages (Kupffer cells), and *Il10* but a lower level of mRNA encoding *Il1b*, an important inflammatory cytokine (Figure 4). No significant differences were found in the expression levels of other cytokines when comparing phage- and vehicle-treated mice following chronic-binge ethanol feeding. These results, together, indicate that *E. faecalis* phage treatment could stimulate an immune response in different parts of the intestine, as well as in the liver.

### 3.2. Phage Translocation

**Figure 5:**
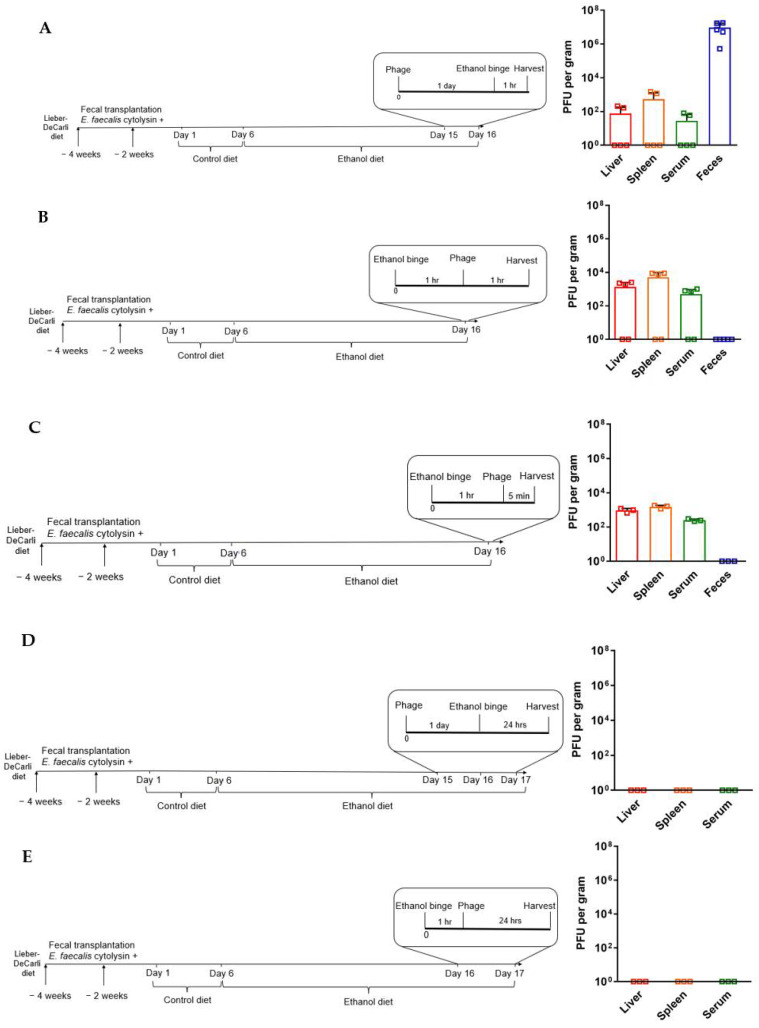
Evaluation of phage translocation. C57BL/6 germ-free mice were colonized with feces from a cytolysin-positive patient with alcohol-associated hepatitis. Mice were then fed with a chronic-binge ethanol diet, (**A**) gavaged with a phage cocktail that targets cytolytic *E. faecalis* (10^10^ PFUs) one day before the ethanol binge, and harvested one hour after the ethanol binge; (**B**) gavaged with a phage cocktail that targets cytolytic *E. faecalis* (10^10^ PFUs) one hour after the ethanol binge, and harvested one hour after the phage administration; (**C**) gavaged with a phage cocktail that targets cytolytic *E. faecalis* (10^10^ PFUs) one hour after the ethanol binge, and harvested 5 min after the phage administration; (**D**) gavaged with a phage cocktail that targets cytolytic *E. faecalis* (10^10^ PFUs) one day before the ethanol binge, and harvested one day after it; (**E**) gavaged with a phage cocktail that targets cytolytic *E. faecalis* (10^10^ PFUs) one hour after the ethanol binge, and harvested one day after the phage administration. *n* = 5 in each group (**A**,**B**); *n* = 3 in each group (**C**–**E**).

Ethanol binge disrupts the gut barrier and promotes microbes to translocate from the intestine to other organs. To assess whether orally administered phages can pass through the gut barrier and reach the systemic circulation, we checked the phage load in the liver, spleen, and serum, as well as in the feces. In the first model, a phage cocktail was administrated one day before the ethanol binge as we had done in the previous section. Mice were euthanized 1 h after the ethanol binge when live phages were detected in the liver, spleen, serum, and feces (Figure 5A). Since patients with alcohol-associated hepatitis have intestinal barrier dysfunction, we determined the phage load under the condition of increased intestinal permeability induced by binge ethanol, administrating the phage cocktail after the ethanol binge. Live phages were detected in the liver, spleen, and serum, but not in the feces (Figure 5B). To further define the kinetics of phage translocation, ethanol binge was done before the phage treatment, and mice were euthanized 5 min following phage gavage. Interestingly, live phages were already detectable in the systemic circulation, liver, and spleen only 5 min following phage administration (Figure 5C), indicating a rapid translocation process in the setting of a disrupted gut barrier. Phages were gavaged either before (Figure 5D) or after (Figure 5E) ethanol binge to determine the clear rate of phages in the systemic circulation. In both models, about 24 h after phages passing through the disrupted gut barrier caused by the ethanol binge, no phages were detected in the serum, liver, and spleen, suggesting a relatively quick clearance in the murine model. Altogether, our results indicate that orally administered phages translocate from the gut to other organs.

### 3.3. Host Range Determination

**Figure 6:**
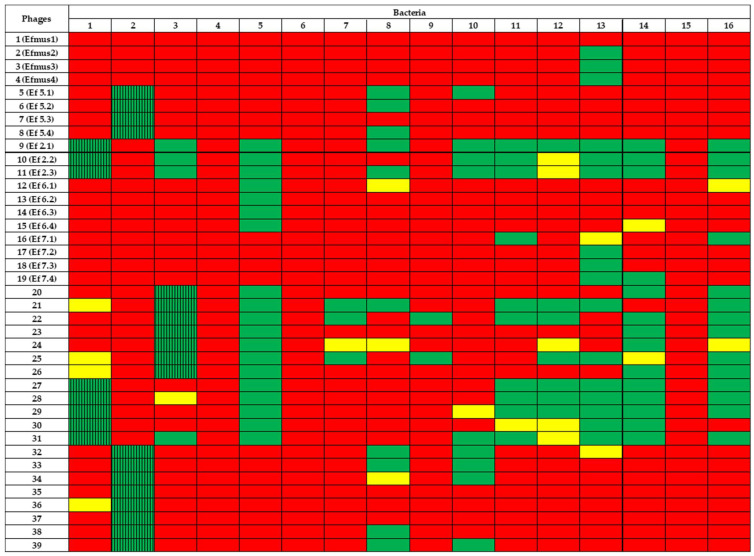
Host range of phages. Each of the isolated *E. faecalis* phages (#1 to #39) was tested on sixteen cytolytic *E. faecalis* strains to determine their host ranges. Phages #1 to #19 have been isolated and characterized [1]. Their original nomenclature was added in parenthesis. Phages #20 to #39 are new isolates. Red: EoP = 0; Yellow: EoP < 0.001; Green: EoP > 0.001. Boxes with lines indicate the bacterial host strain for each phage. EoP: efficiency of plating (PFUs using the specific bacterial strain/PFUs using the original host strain).

We have previously characterized 19 lytic phages with transmission electron microscopy (TEM) images and whole-genome sequencing [1]. Here, we determined the host range of these phages against sixteen cytolytic *E. faecalis* strains, each strain derived from a different patient with alcohol-associated hepatitis. As shown in Figure 6, we found a diversity of host range. In order to find more phages with a possible broader host range, we isolated additional 20 different lytic phages from sewage water (Supplementary Figure S1), using three cytolytic *E. faecalis* strains isolated from the stool samples of patients with alcohol-associated hepatitis as a host. We then determined the host ranges of those newly isolated phages. A total of 13 *E. faecalis* strains were susceptible to at least two of the phages, while three were found to be resistant to all of them. Nine of the 39 phages were found to have a broad host range infecting at least eight of the host strains, but most of them had a narrow host range (less than eight host strains were sensitive).

## 4. Discussion

The therapeutic use of bacteriophages has seen a renewed interest in the last few years, mainly due to the increased occurrence of antibiotic-resistant strains in patients [20]. Recently, we demonstrated that phages against cytolytic *E. faecalis* reduced ethanol-induced liver disease, whereas mice treated with phages against cytolysin-negative *E. faecalis* did not show any beneficial effects, indicating that phage treatment can provide an effective method for precisely editing the intestinal microbiota in patients with alcohol-associated hepatitis, thereby improving clinical outcomes and reducing mortality [1]. Here, in our model of ethanol-induced liver disease in humanized mice, oral treatment with phages against cytolytic *E. faecalis* was able to induce an immune response in the intestine. The upregulation of *Il10, Il17*, and *Il22* in the intestine might indicate that phage treatment has evolved anti-inflammatory and tissue-restoring effects, although we saw a slight upregulation of *Tnfa* in the jejunum. The IL10 family of cytokines (including IL22) is of particular interest for their anti-inflammatory properties and tissue protection, inhibiting the activity of TH1 cells, NK cells and macrophages, and blocking the expression of some inflammatory cytokines [4]. IL17 is a very important mucosal barrier cytokine to defend against invading microbes [21]. Most importantly, we found an increased expression of *Il10* and decreased expression of *Il1b* in the liver following phage treatment. IL1B is a key inflammatory cytokine to mediate alcohol-associated liver disease [22,23]. Several clinical trials have been blocking the IL1B signaling pathway in patients with alcohol-associated hepatitis (ClinicalTrials.gov Identifier: NCT03775109; NCT04072822; NCT01809132). The liver is considered a second firewall for translocating bacteria from the gut [24]. We observed that oral phage treatment can upregulate *F4/80*, a marker of resident Kupffer cells in the liver [25]. Kupffer cells play a crucial role in the phagocytosis of translocated bacteria; thus, an increased number of the first will increase the clearance of the latter [18]. An in vitro study with *Staphylococcus aureus* and *Pseudomonas aeruginosa* phages demonstrated the production of both inflammatory and anti-inflammatory cytokines from peripheral blood mononuclear cells (the prevailing effect being anti-inflammatory) [4]. Moreover, it has been shown that phages can reduce *S. aureus*- or lipopolysaccharide (LPS)-induced levels of *Tnfa, Il1b, Il6,* and *Il10* and suppress the LPS-induced phosphorylation of NF-κB in a model using MAC-T bovine mammary epithelial cells [26].

The question of whether orally administered phages can bypass the intestinal epithelium and migrate to lymph, peripheral blood, and internal organs is equally important, especially from a clinical perspective in patients with alcohol-associated hepatitis [27]. The oral administration of a phage cocktail against cytolytic *E. faecalis* followed by ethanol binge led to a consistent recovery of live phages from serum, spleen, liver, and feces. Since increased intestinal permeability commonly occurs in patients with advanced alcohol-associated liver disease [28,29], we administrated the phage treatment after the ethanol binge as a strategy to mimic this phenotype. In this experiment, increased intestinal permeability induced by the ethanol-binge contributed to the rapid permeation of phages to the spleen, liver, and bloodstream, observed 1 h after phage treatment but also within only five minutes. Conversely, no bacteriophages were recovered about 24 h after passing through the disrupted gut barrier caused by the ethanol binge, suggesting a quick clearance from serum, liver, and spleen. Phage clearance takes place mostly due to the phagocytosis of phage particles in the liver by Kupffer cells and, to a lesser extent, in the spleen by splenic macrophages [20,30]. A disrupted gut barrier is one of the mechanisms proposed that phages utilize to access the body from the intestine [5], allowing them to elicit innate and adaptive immune responses [4]. In the liver, phages can downregulate the expression of cytokines associated with hepatic injury activation (such as Il1b) and upregulate the expression of protective factors (such as Il10). In addition, phages can infect and lyse translocated cytolysin-positive *E. faecalis* in the systemic circulation and liver.

Finally, for therapeutic use in a clinical trial, it is necessary to isolate lytic phages against the bacteria responsible for the patient’s disease and to amplify and administer them in such a way that they can recognize and kill the target bacteria. Here, we report the isolation of 20 new phages targeting three different cytolytic *E. faecalis* strains isolated from fecal samples of patients with alcohol-associated hepatitis. We found a diversity of host range, in which some phages had a quite narrow host range (just a subset of one or two strains), and some of them showed a broader host range. A combination of three phages (#9, #11, and #25) can infect 75% of isolated *E. faecalis* strains. The narrow host range is an apparent limitation of phage-based treatment. Therefore, the superb specificity of phages, which enables the precise targeting of bacteria, is also a potential problem because the narrow host range could limit therapeutic utility [31]. Phage cocktails, the simultaneous use of more than one phage type [32], have shown broad-spectrum activity against many bacterial strains with promising outcomes in both in vitro and in vivo studies [33,34,35,36,37].

In conclusion, here we demonstrate that oral phage administration appears to be safe in an ethanol-induced liver disease mouse model. The translocation of phages to the blood and liver offers the advantage of making it possible for systemic bacteria to be targeted. A cocktail of three lytic phages covers 75% of 16 cytolysin-positive *E. faecalis* isolated from different patients with alcohol-associated hepatitis. However, more studies are needed to validate these findings, and a clinical trial is required to evaluate the phage treatment for patients with alcohol-associated hepatitis.

## Figures and Tables

**Table 1:** List of quantitative PCR primers and their sequence.

Name	Forward	Reverse	Ref.
*18S*	AGTCCCTGCCCTTTGTACACA	CGATCCCAGGGCCTCACTA	[1]
*F4/80*	CATAAGCTGGGCAAGTGGTA	GGATGTACAGATGGGGGATG	[15]
*Foxp3*	CTCGTCTGAAGGCAGAGTCA	TGGCAGAGAGGTATTGAGGG	[16]
*Ifng*	ACAGCAAGGCGAAAAAGGAT	TGCAGTGGGGAAACATGAGAT	[17]
*Il10*	ATCGATTTCTCCCCTGTMAA	TGTCAAATTCATTCATGGCCT	[18]
*Il17A*	TCCAGAAGGCCCTCAGACTA	TGAGCTTCCCAGATCACAGA	[17]
*Il1b*	GGTCAAAGGTTTGGAAGCAG	TGTGAAATGCCACCTTTTGA	[1]
*Il22*	GCTCAGCTCCTGTCACATCA	TCGCCTTGATCTCTCCACTC	[19]
*Tnfa*	AGGGTCTGGGCCATAGAACT	CCACCACGCTCTTCTGTCTAC	[18]

## Data Availability

The data that support the findings of this study are available from the author (B.S.) upon reasonable request.

## Supplementary Materials

The following supporting information can be downloaded at: https://www.mdpi.com/article/10.3390/v14030490/s1, Figure S1: Restriction enzyme digestion. All newly isolated phages were digested by restriction enzymes, then ran on 1% agarose gel, to distinguish different phage isolates, indicated by a different gel pattern. Phages numbers are indicated on the gel.
