# Supplementary material for: Immune Response of an Oral Enterococcus faecalis Phage Cocktail in a Mouse Model of Ethanol-Induced Liver Disease
Source: Viruses. 2022 Feb 27;14(3):490. doi: 10.3390/v14030490 (PMC8955932; doi:10.3390/v14030490)
Supplement: Supplementary file 1 [file viruses-14-00490-s001.zip › viruses-1602568-supplementary.pdf]

Supplementary Figure S1

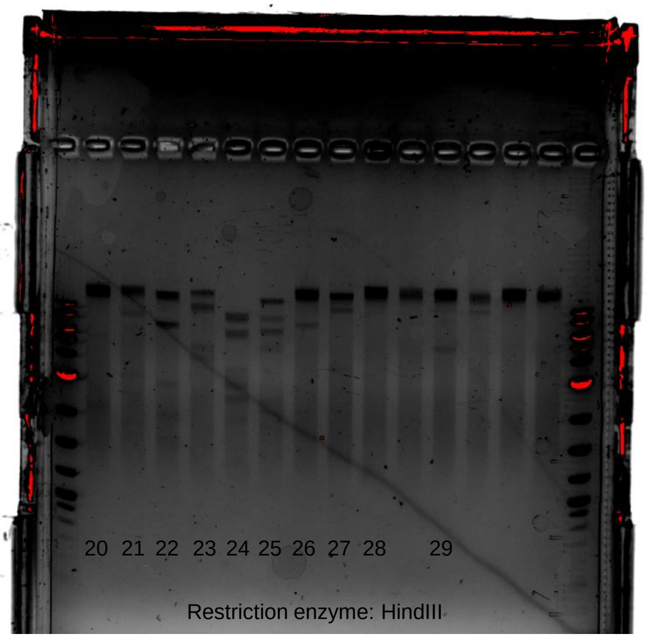

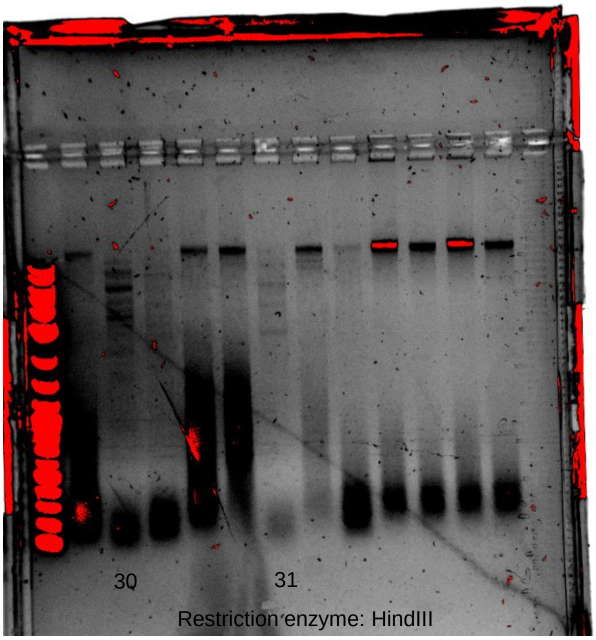

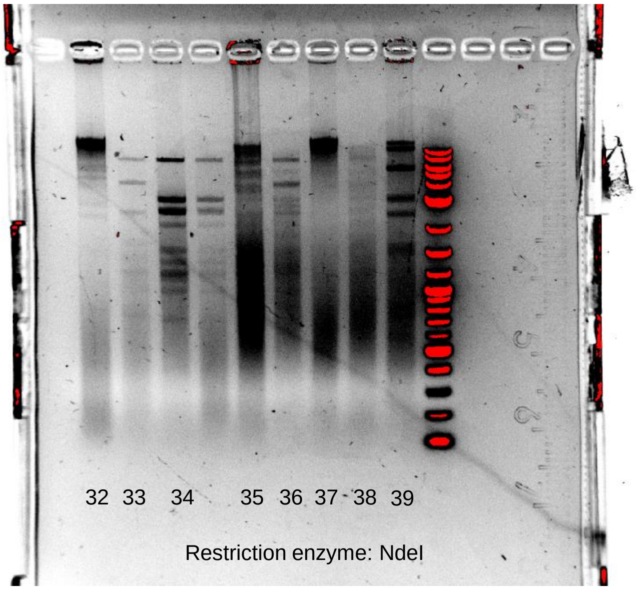

Supplementary Figure S1. Restriction enzyme digestion. All newly isolated phages were digested by restriction enzymes, then ran on 1% agarose gel, to distinguish different phage isolates, indicated by a different gel pattern. Phages numbers are indicated on the gel.
